# Using Linked Administrative Data to Explore Ethnicity, Care Experience and Justice Systems Involvement: Challenges and Possibilities.

**DOI:** 10.23889/ijpds.v7i3.2106

**Published:** 2022-08-25

**Authors:** Katie Hunter

**Affiliations:** 1 University College London (UCL)

The youth and adult criminal justice systems in England and Wales disproportionately draw in individuals from ethnic minority backgrounds (Lammy, 2017; YJB, 2021) and those who have been in care as children (HMIP, 2021; MoJ, 2012). While evidence suggests that there is likely to be considerable overlap between these two groups (Prison Reform Trust, 2016), there was previously little in the way of quantitative data to explore this. The newly linked MoJ/DfE datasets have provided a unique opportunity to investigate the intersections between ethnicity, care experience and justice systems involvement for the first time. This paper will draw on findings from an ADR UK (Administrative Data Research UK) Fellowship utilising the newly linked datasets. It will outline to what extent ethnic minority care-experienced people are over-represented in justice systems. It will also explore the nature of this involvement including offence type, frequency, and disposals. The findings will be contextualised within the wider literature on racialisation (Phillips, 2012) and criminalisation in care (Howard League, 2017) to enable a broader understanding of the issues. The paper will outline the challenges of conducting interdisciplinary research using linked data from across government departments as well as the possibilities for future research.

